# Genome Characterisation of an Isoprene-Degrading *Alcaligenes* sp. Isolated from a Tropical Restored Forest

**DOI:** 10.3390/biology11040519

**Published:** 2022-03-28

**Authors:** Toungporn Uttarotai, Sawannee Sutheeworapong, Andrew T. Crombie, J. Colin Murrell, Wuttichai Mhuantong, Nuttapol Noirungsee, Sunanta Wangkarn, Sakunnee Bovonsombut, Terry J. McGenity, Thararat Chitov

**Affiliations:** 1 Department of Biology, Faculty of Science, Chiang Mai University, Chiang Mai 50200, Thailand; toungporn_u@cmu.ac.th (T.U.); nuttapol.n@cmu.ac.th (N.N.); sakunnee.b@cmu.ac.th (S.B.); 2 School of Life Sciences, University of Essex, Colchester CO4 3SQ, UK; 3 School of Bioresources and Technology, King Mongkut’s University of Technology Thonburi, Bangkok 10150, Thailand; sawannee.sut@kmutt.ac.th; 4 School of Environmental Sciences, University of East Anglia, Norwich NR4 7TJ, UK; a.crombie@uea.ac.uk (A.T.C.); j.c.murrell@uea.ac.uk (J.C.M.); 5 Enzyme Technology Research Team, Biorefinery and Bioproduct Technology Research Group, National Center for Genetic Engineering and Biotechnology, Pathumthani 12120, Thailand; wuttichai.mhu@biotec.or.th; 6 Department of Chemistry, Faculty of Science, Chiang Mai University, Chiang Mai 50200, Thailand; sunanta@chiangmai.ac.th; 7 Environmental Science Research Center (ESRC), Faculty of Science, Chiang Mai University, Chiang Mai 50200, Thailand

**Keywords:** isoprene, climate-active gas, isoprene degradation, genome, *Alcaligenes*

## Abstract

**Simple Summary:**

Isoprene, a volatile hydrocarbon, is the second most abundantly produced climate-active gas, with largely indirect detrimental impacts, such as extending the residence time of the greenhouse gas methane. Isoprene is mainly emitted by plants and can be consumed by a range of microbes inhabiting diverse environments, including soil. Here, the ability of soil bacteria to degrade isoprene was investigated. Soil samples were taken from beneath wild Himalayan cherry trees in a tropical restored forest area, and an *Alcaligenes* sp. (strain 13f) was isolated. This isolate used isoprene as a sole source of carbon and energy (32.6% of isoprene was consumed in 18 days). A surprising finding from the genome analysis of *Alcaligenes* sp. strain 13f was that the well-characterised genes and genetic organisation typical of other isoprene-degrading bacteria were not observed. Thus, we propose that this strain uses a different metabolic pathway for isoprene degradation.

**Abstract:**

Isoprene is a climate-active biogenic volatile organic compound (BVOC), emitted into the atmosphere in abundance, mainly from terrestrial plants. Soil is an important sink for isoprene due to its consumption by microbes. In this study, we report the ability of a soil bacterium to degrade isoprene. Strain 13f was isolated from soil beneath wild Himalayan cherry trees in a tropical restored forest. Based on phylogenomic analysis and an Average Nucleotide Identity score of >95%, it most probably belongs to the species *Alcaligenes faecalis*. Isoprene degradation by *Alcaligenes* sp. strain 13f was measured by using gas chromatography. When isoprene was supplied as the sole carbon and energy source at the concentration of 7.2 × 10^5^ ppbv and 7.2 × 10^6^ ppbv, 32.6% and 19.6% of isoprene was consumed after 18 days, respectively. Genome analysis of *Alcaligenes* sp. strain 13f revealed that the genes that are typically found as part of the isoprene monooxygenase gene cluster in other isoprene-degrading bacteria were absent. This discovery suggests that there may be alternative pathways for isoprene metabolism.

## 1. Introduction

Isoprene (2-methyl-1,3-butadiene; C_5_H_8_) is one of the primary biogenic volatile organic compounds [1]. It is emitted into the atmosphere by many organisms, especially plants [2]. It is estimated that global emissions of isoprene are in the range of 400 to 600 Tg per year, comparable in scale to methane [3,4]. Isoprene plays an important role in the Earth’s climate by reacting with free radicals in the atmosphere to produce greenhouse gases or by extending the residence time of other gases such as methane, thereby resulting in increased global temperatures [5].

Previous studies have shown that freshwater, marine, and soil ecosystems, and the phyllosphere, all operate as sinks for isoprene [6,7,8,9,10,11,12]. For example, it has been estimated that soil bacteria can consume up to 20.4 Tg of isoprene per year [7,10]. Many isoprene-degrading bacteria have been isolated from diverse environments, such as *Arthrobacter*, *Nocardia*, *Nocardioides*, *Rhodococcus*, *Gordonia*, *Bacillus*, *Ramlibacter*, *Variovorax*, *Klebsiella*, *Mycobacterium*, *Pseudomonas*, and *Alcaligenes* [7,8,9,13,14,15,16,17,18,19]. Some of these isoprene-degrading bacteria have been genetically characterised, including Gram-negative bacteria, such as *Variovorax*, *Ramlibacter*, and *Sphingopyxis* [18,19], and Gram-positive bacteria, such as *Rhodococcus*, *Nocardioides*, *Gordonia*, and *Mycobacterium* [15,16,17,20]. Among these, *Rhodococcus* AD45, a freshwater sediment strain, is probably the best-characterised isoprene-degrading bacterium and has been used as a model for isoprene metabolism studies [16,20].

Among those isoprene-degrading bacteria that have been analysed, there is a high level of genetic conservation. All of the genomes of isoprene degraders that have been sequenced harbour a gene cluster (*iso* cluster) that encodes the enzymes required for isoprene degradation. Some genes in the cluster (*isoABCDEF*) encode a multi-subunit isoprene monooxygenase that catalyses the oxidation of isoprene to epoxyisoprene, whereas others (*isoGHIJ*) encode a glutathione transferase and enzymes involved in subsequent stages of isoprene oxidation [16,21,22]. This genetic characterisation has led to the development of molecular methods to determine isoprene-degrading capability through PCR amplification of the *isoA* gene, the gene encoding the putative active site of isoprene monooxygenase which catalyses the first step in isoprene degradation. In all isoprene-degrading bacterial strains that were genetically examined, this gene has been found, thus making it a suitable molecular target to investigate the diversity and abundance of isoprene degraders [14,15,23]. However, since the methods were designed based on a relatively limited number of isoprene-degrading bacterial genera [14,23], it is not known if *isoA* is found in all isoprene degraders, or whether there are alternative mechanisms of isoprene degradation.

One of the most environmentally significant bacterial genera that has been the least studied in relation to isoprene degradation is *Alcaligenes*. It is a Gram-negative bacterium that is ubiquitous in the natural environment, such as soil, fresh waters, marine environments, and industrial effluent [24,25,26,27]. This genus was first described in 1919 [28] and has had a wide range of applications in bioremediation because of its ability to degrade many pollutants such as phenols, DDT insecticide, and hydrocarbons in crude oil [27,29,30]. In relation to isoprene, *Alcaligenes* was first reported as an isoprene degrader in 1990 [31]. Later, in 2015, a species of *Alcaligenes* was isolated from contaminated soil taken from a waste rubber dumping site and was confirmed for its ability to utilise isoprene as the sole source of carbon and energy [32]. However, these reports on isoprene-degrading *Alcaligenes* species did not provide characterisation at the biochemical or molecular biological level.

In this study, an *Alcaligenes* strain was isolated from tropical restored forest soil in Doi Suthep-Pui National Park in the north of Thailand. The strain was associated with wild Himalayan cherry, one of the indigenous tree species that has been recommended as a framework species for forest restoration [33]. Many isoprene-degrading bacteria, including *Ochrobactrum*, *Arthrobacter*, *Bacillus*, *Friedmanniella*, *Klebsiella*, *Isoptericola*, and *Cellulosimicrobium*, had been found in this location [34]. Here, we investigated the metabolism of isoprene by this *Alcaligenes* strain through its genome characterisation.

## 2. Materials and Methods

### 2.1. Enrichment and Isolation of Isoprene-Degrading Bacteria

The isoprene-degrading bacterium *Alcaligenes* sp. strain 13f was isolated from topsoil (3 cm from the surface) beneath wild Himalayan cherry trees (*Prunus cerasoides* D. Don) in a restored forest area (18°51′51″ N 98°50′51″ E) located in Doi Suthep-Pui National Park, Chiang Mai, Thailand. A one-gram portion of the soil sample was suspended in 9 mL of minimal medium (composition described by Uttarotai et al. [34]) in a vial (125 cm^3^) and tightly closed with PTFE/silicone septum. Isoprene from Sigma-Aldrich (St Louis, MO, USA) (7.2 × 10^5^ ppbv) was injected into the bottle containing the soil sample (isoprene for injection was prepared according to the method described by Acuña Alvarez et al. [13]). After incubation at 27 °C for 96 h, the enriched sample (1 mL) was drawn and subjected to serial dilution and spread-plating on minimal medium agar. The plates were incubated at 27 °C in a glass desiccator with the presence of isoprene (1 mL of isoprene was added to the 10.5 L desiccator). The representatives of colonies of the predominant bacteria grown on minimal medium agar were restreaked and incubated under the same condition until pure cultures were obtained.

### 2.2. Initial Identification of Isoprene-Degrading Bacteria by 16S rRNA Gene Sequencing

Genomic DNA was extracted by using a phenol-chloroform extraction method [35]. The bacterial universal primers (final concentration of 0.4 μM) 27F (5′-AGAGTTTGATCMTGGCTCAG-3′) and 1492R (5′-CGGTTACCTTGTTACGACTT-3′) [36], together with the AppTaq RedMix (Appleton, Birmingham, UK) reaction mixture, were used to amplify the 16S rRNA gene. The PCR was performed with an initial denaturation step at 95 °C for 3 min, 30 cycles of denaturation at 95 °C for 15 s, annealing at 55 °C for 15 s, extension at 72 °C for 30 s, and a 5-min final extension at 72 °C. The PCR products were purified by using a PCR purification kit (Sigma; GenElute, St Louis, MO, USA). Sanger sequencing was performed by Eurofins Genomics (Ebersberg, Germany). Chromas software (version 2.6.6; Technelysium, South Brisbane, Australia) was used to trim low-quality sequences and Bioedit (version 7.05.3) [37] was used to merge forward and reverse DNA sequences. The 16S rRNA sequence was subjected to BLASTn to identify the closest relatives. An isolate (13f), which was identified as Alcaligenes, was selected for further study. The sequence was deposited into the NCBI database (accession number MZ323998).

### 2.3. Test for Isoprene Degradation by Bacterial Isolates

The ability of *Alcaligenes* sp. strain 13f to degrade isoprene was investigated. *Rhodococcus* sp. strain bl28ba, a known isoprene degrader isolated by Murphy [38], was included as a positive control. A loopful of the bacterial culture, grown on minimal medium agar supplied with isoprene (as described in Section 2.1), was resuspended in 100 µL of minimal medium, and transferred to 9.9 mL of minimal medium without isoprene, minimal medium with isoprene, and glucose/yeast-extract broth [34], also with isoprene. The media were contained in 125 cm^3^ vials (as above). For each enrichment, isoprene was supplied at 7.2 × 10^5^ ppbv and 7.2 × 10^6^ ppbv (achieved by injecting 0.1 mL and 1 mL of headspace isoprene gas (prepared as described above) into the vials, respectively). Every three days, bacterial growth was determined by OD_600_, measured by using a Jenway 7300 spectrophotometer (Jenway, Stone, UK), and residual isoprene concentrations were measured by using a Unicam 610 gas chromatograph (Unicam, Lisbon, Portugal) with a flame ionisation detector (FID) and a 10% Apiezon L CW column, following the conditions described by Uttarotai et al. [34].

After 18 days of incubation, the cultures were diluted, spread on minimal medium agar, and incubated in a glass desiccator containing isoprene (as described in Section 2.1) for a further 18 days to confirm the presence of active bacteria and the purity of the bacterial cultures.

### 2.4. Amplification of the isoA Gene

PCR amplification of *isoA* was carried out by using two different protocols. The first protocol employed the *isoA* primers of El Khawand et al. [14] (5′-TGCATGGTCGARCAYATG-3′ and 5′-GRTCYTGYTCGAAGCACCACTT-3′), which yield an expected amplicon of 1015 bp. This protocol was used with a touchdown PCR, starting with an initial denaturation at 94 °C for 3 min, followed by 19 cycles of 94 °C for 30 s, 72 °C for 45 s with a reduction in temperatures of 1 °C per cycle until reaching 54 °C, 72 °C for 60 s, then 25 cycles of 94 °C for 30 s, 54 °C for 45 s, 72 °C for 60 s, and a final extension at 72 °C for 5 min. The other PCR was performed with primers isoA14F (5′-GVGACGAYTGGTAYGACA-3′) and isoA511R (5′-TCGTCRAAGAARTTCTTBAC-3′) of Carrión et al. [23], which yield an expected amplicon of 497 bp. The PCR consisted of an initial denaturation of 94 °C for 2 min, followed by 31 cycles of 95 °C for 15 s, 54 °C for 30 s, 72 °C for 1 min, and a final extension at 72 °C for 7 min. Both reactions were performed by using AppTaq RedMix (Appleton, Birmingham, UK). The PCR products were examined on an agarose gel stained with ethidium bromide.

### 2.5. Preparation of Genomic DNA for Genome Analysis

Genomic DNA from *Alcaligenes* sp. strain 13f was extracted from the culture grown in glucose/yeast-extract broth at 20 °C for 5 days by using the GenElute Bacterial Genomic DNA Extraction Kit (Sigma, St Louis, MO, USA). The DNA was examined on an agarose gel and quantified by using the Quant-iT PicoGreen dsDNA assay (Thermo Scientific, Loughborough, UK) in a Nanodrop spectrophotometer (NanoDrop 3300, Thermo Scientific, Loughborough, UK).

### 2.6. Genome Sequencing and Genome Assembly

The genome of *Alcaligenes* sp. strain 13f was sequenced by MicrobesNG (University of Birmingham, Birmingham, UK). Genomic DNA libraries were prepared by using a Nextera XT Library Prep Kit (Illumina, San Diego, CA, USA) according to the manufacturer’s protocol with the following modifications: two nanograms of DNA were used, and the PCR elongation time was 1 min. DNA quantification and library preparation were carried out by using a Hamilton Microlab STAR automated liquid handler. Pooled libraries were quantified by using the Kapa Biosystems Library Quantification Kit for Illumina on a Roche LightCycler 96 qPCR machine. Libraries were sequenced on the Illumina HiSeq by using a 250-bp paired-end protocol. Reads were adapter-trimmed by using Trimmomatic (version 0.30) [39] with a sliding window quality cutoff of Q15. De-novo assembly was performed by using SPAdes (version 3.7) [40] with default parameters.

### 2.7. Phylogenomic Characterisation and Analysis of Average Nucleotide Identity (ANI)

Twelve publicly available genomes of Alcaligenes spp. were included for phylogenomic analysis. The Pathosystems Resource Integration Center (PATRIC) [41] was used to construct a codon-based tree built on 500 single-copy genes that are present in all genomes studied (Table S1), and aligned with the MAFFT (version 7.397) [42]. The maximum likelihood method (Jones-Taylor-Thornton (JTT) model) was performed by using RaxML (version 8.2.11) [43] with branch support values determined by 100 replicates of fast bootstrapping. The phylogenomic tree was visualised by using iTOL [44].

The similarities of eight genomes of closely related *Alcaligenes* species, including *Alcaligenes* sp. strain 13f, were analysed based on Average Nucleotide Identity (ANI) values, which were calculated and visualised by using OAT (version 0.93.1) [45].

### 2.8. Genome Analysis and Comparison

The quality of the assembly was assessed by using QUAST (version 5.0.2) [46] and sequences shorter than 200 bp were removed before the annotation process. The completeness of the genome assembly was determined by using Benchmarking Universal Single-Copy Orthologs (BUSCO) (version 4.1.3) [47]. The annotation was done by using the NCBI Prokaryotic Genome Annotation Pipeline (PGAP) (version 5.2) (with the best-placed reference protein set and GeneMarkS-2+ methods) [48]. The amino acid sequences from *Alcaligenes* sp. strain 13f were compared with those of known bacterial isoprene degraders by using BLASTp (version 2.12.0) [49] to search for the occurrence of enzymes involved in isoprene degradation. To search for homologues of specific amino acid sequences, a local nucleotide BLAST database was queried with amino acid sequences of interest by using tBLASTn (version 2.2.10) [49]. The genomic data from this study have been deposited in the NCBI database under the accession number PRJNA734706.

## 3. Results and Discussion

### 3.1. Growth Characteristics and Isoprene Degradation of Alcaligenes sp. Strain 13f

Isoprene-degrading bacteria were isolated from isoprene-enriched soil samples collected beneath wild Himalayan cherry trees. A representative (isolate 13f) of the white, opaque colonies on minimal medium incubated with isoprene, which were the majority of colonies obtained, was further characterised and identified. It was Gram-negative, rod-shaped, identified as being related to *Alcaligenes faecalis*, according to 16S rRNA gene sequencing. Strain 13f used isoprene as the sole source of carbon and energy, as demonstrated by the growth on minimal medium agar incubated under isoprene as the sole carbon source.

**Figure 1:**
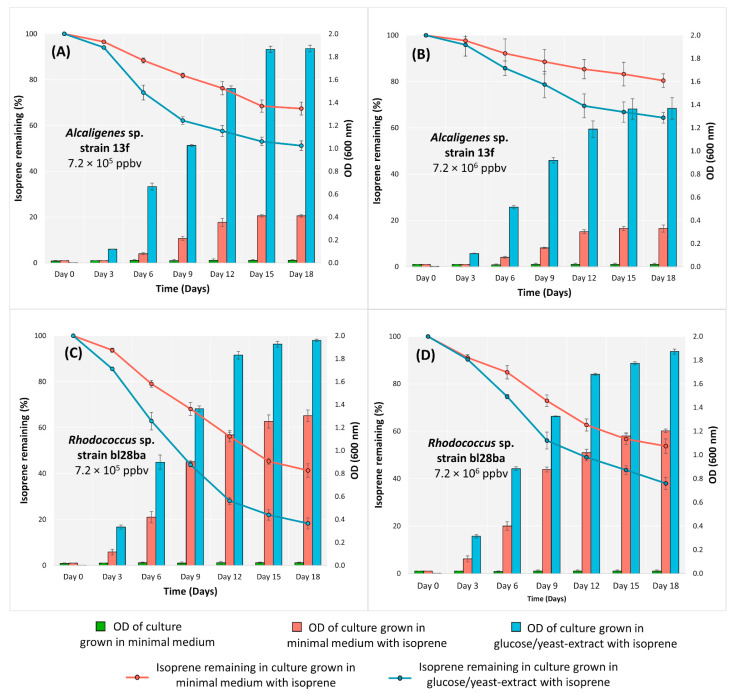
Growth and isoprene degradation by *Alcaligenes* sp. strain 13f (**A**,**B**) and *Rhodococcus* sp. strain bl28ba (**C**,**D**) in minimal medium and glucose/yeast-extract medium incubated with isoprene at two different concentrations over 18 days. Growth of each isolate was demonstrated by an increase in OD_600_, and isoprene degradation is shown by the percentage of remaining isoprene in the culture, *n* = 3. The error bars show ± SD.

*Alcaligenes* sp. strain 13f was then grown in minimal medium broth supplemented with isoprene at two concentrations (7.2 × 10^5^ and 7.2 × 10^6^ ppbv). The degradation of isoprene from the treatment with the lower isoprene concentration was 32.6% (Figure 1A, pink line), greater than that from the treatment with the higher isoprene concentration, which was 19.6% (Figure 1B, pink line) (Anova; *p* < 0.05).

When *Alcaligenes* sp. strain 13f was additionally provided with glucose/yeast extract, it degraded isoprene more rapidly and to a greater extent than when isoprene was the only source of carbon and energy (Figure 1A,B, blue lines) (Anova; *p* < 0.05). It also grew more rapidly and extensively in the presence of glucose/yeast extract (Figure 1A,B, blue bars) (Anova; *p* < 0.05). These findings indicate that the genes for isoprene degradation are not repressed by the alternative organic growth substrates in the medium, as seen in other strains [34,50].

Similar to the cultures grown in minimal medium, in glucose yeast-extract broth, the extent of isoprene degradation was significantly lower (Anova; *p* < 0.05) when incubated at the higher isoprene concentration. Isoprene degradation in the glucose/yeast-extract broth after 18 days was 48.8% in the treatment supplied with the lower isoprene concentration (Figure 1A, blue line) and 35.6% with the higher isoprene concentration (Figure 1B, blue line).

An *Alcaligenes* sp. was previously described as an isoprene degrader by Ewers et al. [31]. Srivastva et al. [32] also observed the ability of an *Alcaligenes* sp. isolated from waste rubber dumping site soil to degrade isoprene and found that isoprene degradation by *Alcaligenes* strain ISO1 decreased with increasing isoprene concentrations, perhaps indicative of isoprene toxicity at higher concentrations.

The isoprene degradation and the growth of *Alcaligenes* sp. strain 13f were higher than those of the bacteria that had previously been reported, which were *Ochrobactrum*, *Arthrobacter*, *Bacillus*, *Friedmanniella*, *Klebsiella*, *Isoptericola*, and *Cellulosimicrobium* genera [34]. However, the growth and isoprene degradation by *Alcaligenes* sp. strain 13f (Figure 1A,B) were lower than those of *Rhodococcus* sp. strain bl28ba in every treatment (Figure 1C,D). *Rhodococcus* is a commonly isolated isoprene-degrading species in soil enriched with isoprene [14,23].

### 3.2. Examination of isoA Gene in Alcaligenes sp. Strain 13f

The presence of the *isoA* gene, encoding the isoprene monooxygenase subunit and currently considered as an essential component of the pathway for isoprene degradation, was examined in *Alcaligenes* sp. strain 13f by using the primers designed by El Khawand et al. [14] and Carrión et al. [23]. *Rhodococcus* sp. strain bl28ba was used as a positive control. No *isoA* product was amplified by PCR from *Alcaligenes* sp. strain 13f, which led to the hypothesis that the *isoA* sequence from *Alcaligenes* sp. strain 13f was very different from the *isoA* sequences from diverse isoprene-degrading genera: *Gordonia*, *Leifsonia*, *Loktanella*, *Micrococcus*, *Mycobacterium*, *Nocardioides*, *Rhodococcus*, *Shinella*, *Sphingopyxis*, *Stappia*, and *Variovorax* [14,23]. Another hypothesis was that *Alcaligenes* sp. strain 13f lacks the *isoA* gene and has a novel mechanism of isoprene degradation. Therefore, to start to address these hypotheses, the genome of *Alcaligenes* sp. strain 13f was sequenced.

### 3.3. Overall Characteristics of the Genome of Alcaligenes sp. Strain 13f

The genome of *Alcaligenes* sp. strain 13f was 4,402,996 base pairs, which is within the size range for this genus (3.02–4.86 Mbp). The mol% GC content of 56.29% was also typical of this genus. Other genomic information is described in Table 1.

According to its 16S rRNA gene sequence from the genome sequencing, strain 13f was identified as *Alcaligenes faecalis*, and the closest relative was strain NBRC 13111 with an identity of 99.73%.

**Figure 2:**
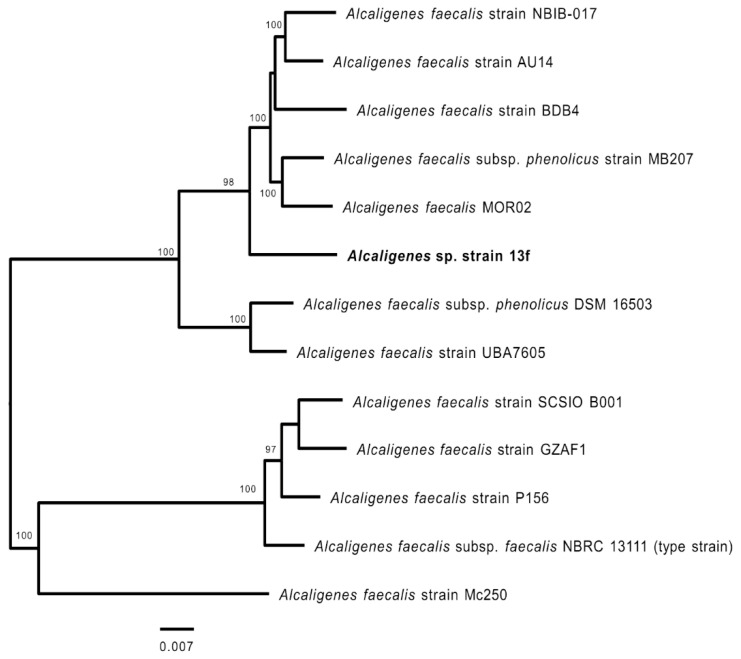
Phylogenomic analysis of *Alcaligenes* spp. including *Alcaligenes* sp. strain 13f (bold font). The tree shown is a maximum likelihood phylogeny of 500 genes (presented in Table S1), based on 100 rounds of rapid bootstrapping. Only the bootstraps supporting values of >50% are shown.

To investigate the phylogenomic relationship of *Alcaligenes* sp. strain 13f, its genome sequence was uploaded to the PATRIC workspace [41]. In total, 500 single-copy genes that are commonly present in the genomes of 12 strains of *Alcaligenes* spp., which were selected based on previously described 16S rRNA phylogeny, were used to build a phylogenomic tree. Strain 13f was shown to most likely be a member of the species *Alcaligenes faecalis* (Figure 2). The closest strains were AU14, NBIB-017, BDB4, MB207, and MOR02 (Figure 2), and included members of the subspecies *phenolicus*, whereas 16S rRNA gene sequence analysis identified the closest strain as NBRC 13111, the type strain.

**Figure 3:**
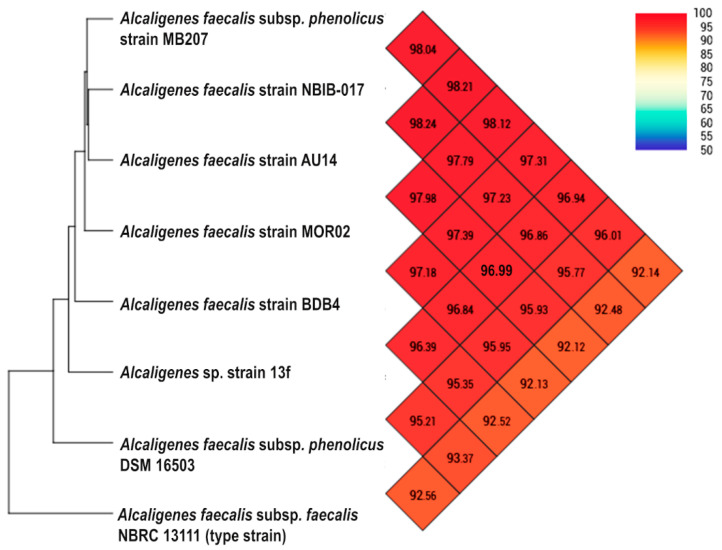
A heatmap presenting the ANI values of *Alcaligenes* sp. strain 13f and seven closely related strains, including the type strain (*Alcaligenes faecalis* subsp. *faecalis* NBRC 13111). The heatmap was calculated by using the Orthologous Average Nucleotide Identity Tool (OAT) software.

To confirm the species assignment based on phylogenomic results, we applied one of the most effective measures for determining the relationships between bacterial species based on genome comparison, namely Average Nucleotide Identity (ANI) [51]. Analysis of the genome of *Alcaligenes* sp. strain 13f compared with the genomes of six of the most closely related strains and the type strain (*Alcaligenes faecalis* subsp. *faecalis* NBRC 13111) revealed that all of the ANI values were higher than 92% (Figure 3). If the type strain (NBRC 13111) is excluded, *Alcaligenes* sp. strain 13f and each of the other six strains had ANI values of more than 95% (the threshold for shared species identity [52,53]). The highest ANI value was 96.99% identity with *Alcaligenes faecalis* strain AU14, a wheat root strain with the ability to reduce nitrous oxide [54]. Thus, the phylogenomic circumscription of strain 13f by strains of *Alcaligenes faecalis* and the high genome relatedness based on ANI suggest that it is probably a member of this species.

### 3.4. Genome Comparison between Alcaligenes sp. Strain 13f and Other Isoprene-Degrading Bacteria

In order to identify the sequences that might code for genes involved in isoprene degradation, the genes from the genome of *Alcaligenes* sp. strain 13f were submitted to the NCBI database for annotation by using the prokaryotic genome annotation pipeline (PGAP). Out of the 3942 gene products analysed, seven were found to have some similarities (ca. 20–30%) to proteins previously known to be involved in isoprene degradation in other species (Table 2).

**Figure 4:**
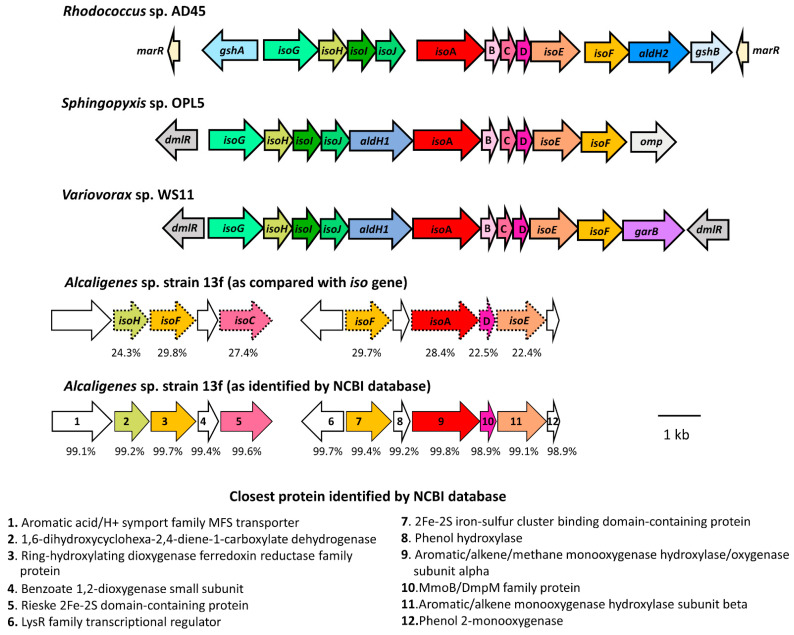
The organisation of the genes putatively involved in isoprene degradation in *Alcaligenes* sp. strain 13f, presented in comparison with the *iso* gene organisation in the reference isoprene degraders: *Rhodococcus* AD45, *Sphingopyxis* OPL5, and *Variovorax* WS11 [18,19,20]. The arrows represent the orientation of the genes. The dotted frames surrounding the gene names are to remind the reader of the putative functional homologues involved in isoprene degradation, with the percent identity to the protein sequences of *Variovorax* WS11 shown below. The bottom-row diagram shows the gene arrangement in *Alcaligenes* sp. strain 13f according to the closest proteins identified by NCBI protein database.

Specifically, the amino acid sequences of *Alcaligenes* sp. strain 13f were compared with the translated sequences from the *iso* gene cluster of *Rhodococcus* AD45, *Sphingopyxis* OPL5, and *Variovorax* WS11, which are isoprene degraders that have been well characterised genetically [18,19,20]. The translated amino acid sequences of *Alcaligenes* sp. strain 13f had some similarities with several proteins encoded in the *iso* metabolic gene cluster found in these strains. However, the percent identities to the Iso sequences were not as high as expected, in the range of approximately 20 to 30% (Table 2). These sequences from *Alcaligenes* sp. strain 13f were identified as other proteins, with more than 98% identities (Table 2). For example, the sequence that had approximately 28% identity to the IsoA sequences in *Rhodococcus* AD45, *Sphingopyxis* OPL5, and *Variovorax* WS11 was identified as having 99.80% identity to an aromatic/alkene/methane monooxygenase hydroxylase/oxygenase subunit alpha from *Alcaligenes* (Table 2). Moreover, some genes typically found together in the *iso* cluster were missing (Figure 4), including *isoB* (encoding isoprene monooxygenase gamma subunit), *isoG* (encoding a putative coenzyme-A transferase), *isoI*, and *isoJ* (encoding a glutathione S-transferase). The absence of these genes indicated that *Alcaligenes* sp. strain 13f might have had a different oxygenase from the previously known isoprene monooxygenase, which enabled it to catabolise isoprene, albeit less rapidly than most well-characterised isoprene-degrading strains [15,16,17,18,19,20].

**Figure 5:**
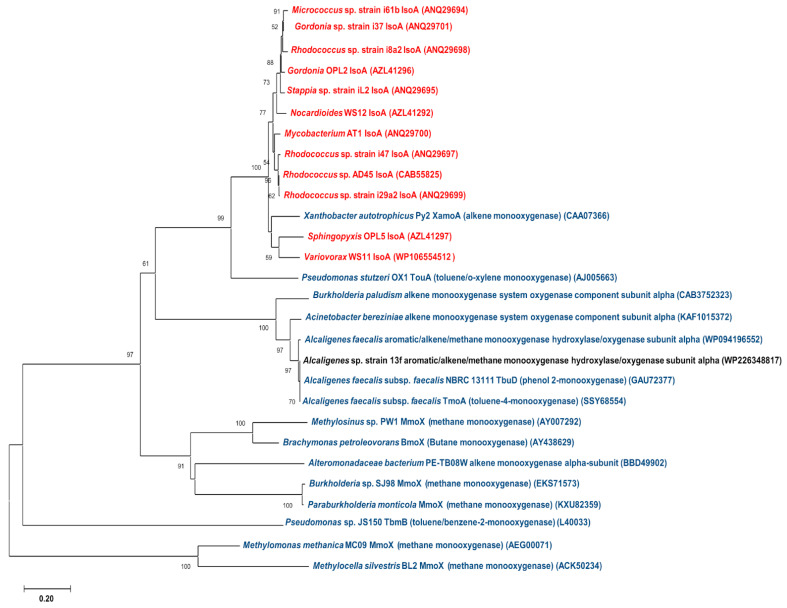
A phylogenetic tree demonstrating the similarities among the IsoA amino acid sequences of known isoprene-degrading strains (red), monooxygenase of *Alcaligenes* sp. strain 13f (black), and other hydrocarbon monooxygenases of other bacteria (blue). A tree was constructed through the maximum composite likelihood model of neighbour-joining method with 1000 bootstrap replicates.

Because a monooxygenase (aromatic/alkene/methane monooxygenase hydroxylase/oxygenase subunit alpha) that had some degrees of similarity to IsoA was found in *Alcaligenes* sp. strain 13f (as seen in Table 2), its amino acid sequence was compared with other *isoA* sequences of selected known isoprene degraders and other monooxygenases, such as methane monooxygenase, toluene monooxygenase, and phenol monooxygenase, by using Bioedit sequence alignments [37]. From the comparison, a phylogenetic tree was constructed using MEGA-X [55] (Figure 5).

The phylogenetic tree revealed notable distances between the monooxygenase found in *Alcaligenes* sp. strain 13f and the *isoA* gene of other isoprene degraders (Figure 5). It can also be seen that the IsoA sequences of Gram-positive bacteria (*Micrococcus*, *Gordonia*, *Rhodococcus*, *Stappia*, *Nocardioides*, and *Mycobacterium*) were phylogenetically grouped, and separate from the group of IsoA sequences of Gram-negative bacteria (*Sphingopyxis* and *Variovorax*), which were closely related to the alkene monooxygenase alpha subunit (*xamoA*) of the Gram-negative *Xanthobacter* strain Py2 (Figure 5), as previously reported by Dawson et al. [18]. The aromatic/alkene/methane monooxygenase found in *Alcaligenes* sp. strain 13f, however, was in a different cluster from both of these groups, and was much more similar to other hydrocarbon monooxygenases (e.g., phenol monooxygenase, alkene monooxygenase, and toluene monooxygenase) from other *Alcaligenes* strains and some other Gram-negative bacteria. The low similarities between the monooxygenases of *Alcaligenes* sp. strain 13f and the proteins in the Iso cluster found in other isoprene degraders are consistent with the lack of detection of the *isoA* gene in *Alcaligenes* sp. strain 13f by PCR (see Section 3.2).

Due to the lack of close homologues of isoprene monooxygenase, and the possibility that a different class of monooxygenase might catalyse the initial oxidation of isoprene, the genome of *Alcaligenes* sp. strain 13f was searched for another key gene of isoprene degradation, *isoI*, encoding the gluthathione-S-transferase which catalyses the second step of the isoprene degradation pathway. This gene is universally present in all previously sequenced isoprene degraders [6]. Since recently, an analogous pathway of styrene degradation has been identified in *Gordonia*, whereby styrene oxide, formed by the action of a flavin-dependent monooxygenase (StyAB), is similarly conjugated with glutathione by StyI [56], the closely-related *styI* gene that was included in the search. When the *Alcaligenes* sp. strain 13f genome was searched for *isoI* or *styI* sequences, no close homologues were found (no hits at E-value < 0.00001). Because the styrene degradation pathway may be non-specific, the *Alcaligenes* sp. strain 13f genome was also searched for homologues of the gene encoding styrene monooxygenase large subunit, *styA*, using as query the amino acid sequences from styrene degraders *Pseudomonas* sp. VLB120 and *Gordonia rubripertincta* CWB2 [56,57]. An FAD-binding oxidoreductase (WP_226349106.1) was identified at a relatively low level of amino acid identity (29% for each query sequence). Because close homologues of WP_226349106.1 are present in many other *Alcaligenes* and related bacteria not known for isoprene degradation, and downstream genes involved in isoprene or styrene degradation were not located nearby in the genome (phenylacetaldehyde dehydrogenase (*styD*), styrene oxide isomerase (*styC*), or glutathione-S-transferase (*isoI*, *styI*)), this was not considered a likely candidate enabling growth on isoprene in *Alcaligenes* sp. strain 13f.

Interestingly, the growth and isoprene degradation of *Alcaligenes* sp. strain 13f were lower than those of *Rhodococcus* sp. strain bl28ba (a representative *Rhodococcus* strain harbouring the *isoA* gene [38], which was used as a positive control) (Figure 1). This was also another indication that the genetics and the mechanisms involved in isoprene metabolism of *Alcaligenes* sp. strain 13f might be different from the typical *iso*-driven mechanisms found in *Rhodococcus* and other genera such as *Gordonia* and *Variovorax* [15,16,18,20], which can contribute to the differences in its pattern of growth and isoprene degradation. However, the mechanism of isoprene catabolism by *Alcaligenes* sp. strain 13f is not yet known but certainly warrants further investigation. The aromatic/alkene monooxygenase found in *Alcaligenes* sp. strain 13f might catalyse the initial oxidation of isoprene, resulting in the decrease of isoprene from the headspace gaseous content. It has been shown previously that aromatic monooxygenases can catalyse the oxidation of alkenes [58]. The resulting products of this catalysis could enter the central metabolic pathway through beta-oxidation, similar to the known isoprene degradation pathway of *Rhodococcus* AD45. Further studies on the intermediate metabolites, as well as transcriptomic/proteomic responses to growth on isoprene compared with other carbon and energy sources, are therefore crucial in deciphering the isoprene degradation pathway of this *Alcaligenes* isolate and *Alcaligenes* spp. in general.

## 4. Conclusions

In this study, *Alcaligenes* sp. strain 13f obtained from soil associated with wild Himalayan cherry was investigated for its isoprene-degrading ability, and its genome was sequenced. Overall, our study showed the capacity for isoprene degradation of an *Alcaligenes* species and raised the intriguing possibility that the isoprene metabolism pathway of this bacterium might be different from other isoprene-degrading bacteria found in previous studies, as revealed by the genome analysis. The incompleteness of the isoprene monooxygenase gene cluster in isoprene-degrading bacteria has never been reported previously. This study, therefore, provides a basis for further exploration into isoprene degradation mechanisms in *Alcaligenes* to gain a better understanding of isoprene metabolism in this environmentally significant isoprene-degrading bacterium.

## Figures and Tables

**Table 1:** The characteristics of the genome of *Alcaligenes* sp. strain 13f.

Genomic Information of *Alcaligenes* sp. Strain 13f	Value
Total length (bp)	4,402,996
GC content (%)	56.29
Number of contigs	86
Largest contig (bp)	320,621
N50 (bp)	180,050
Completeness (%)	100
Protein coding sequences	3942
rRNAs (5S, 16S, 23S)	1, 1, 1
tRNAs	53
ncRNAs	4
Pseudogenes (total)	38

**Table 2:** Percentages of protein sequence identity derived from *Alcaligenes* sp. strain 13f compared with proteins in the Iso metabolic cluster of isoprene-degrading bacteria.

*Alcaligenes* sp. Strain 13f Protein Sequence Accession No.	Closest Protein Identified by BLASTp (NCBI Database) (>98% Identity)	Closest Protein in Iso Cluster	% Identity to Amino Acid Sequence in Iso Cluster of Isoprene Degrader		
			*Rhodococcus* AD45	*Sphingopyxis* OPL5	*Variovorax* WS11
WP_226348813	1,6-dihydroxycyclohexa-2,4-diene-1-carboxylate dehydrogenase	IsoH	-	23.98	24.29
WP_226348814	Ring-hydroxylating dioxygenase ferredoxin reductase family protein	IsoF	30.36	28.97	29.75
WP_226348815	2Fe-2S iron-sulfur cluster binding domain-containing protein	IsoF	29.08	29.46	29.68
WP_226348817	Aromatic/alkene/methane monooxygenase hydroxylase/oxygenase subunit alpha	IsoA	27.77	28.09	28.36
WP_003800437	MmoB/DmpM family protein	IsoD	23.94	26.77	22.54
WP_226348818	Aromatic/alkene monooxygenase hydroxylase subunit beta	IsoE	24.2	20.18	22.36
WP_226348895	Rieske 2Fe-2S domain-containing protein	IsoC	-	-	27.42

## Data Availability

The 16S rRNA sequence and the genomic data are available on the NCBI database (accession number MZ323998 and PRJNA734706).

## Supplementary Materials

The following supporting information can be downloaded at: https://www.mdpi.com/article/10.3390/biology11040519/s1, Table S1: Genes of *Alcaligenes* sp. strain 13f and the other twelve *A. faecalis* strains included in phylogenomic analysis.
