# Supplementary material for: Genome Characterisation of an Isoprene-Degrading Alcaligenes sp. Isolated from a Tropical Restored Forest
Source: Biology (Basel). 2022 Mar 28;11(4):519. doi: 10.3390/biology11040519 (PMC9030188; doi:10.3390/biology11040519)
Supplement: Supplementary file 1 [file biology-11-00519-s001.zip › biology-1623876-supplementary.pdf]

Genome Characterisation of an Isoprene-Degrading *Alcaligenes* sp. Isolated from a Tropical Restored Forest

In order to analyse the relatedness of *Alcaligenes faecalis* strain 13f to other strains of *Alcaligenes faecalis*, twelve genomes of several strains of *Alcaligenes faecalis* available in the NCBI database were used to construct a phylogenomic tree. The chosen strains were closely related to *Alcaligenes faecalis* strain 13f according to their 16S rRNA gene sequences. In total, 500 genes were included for phylogenomic analysis. These genes are part of the core genomes, which are conserved in all of the analysed strains. The list of the genes is shown in Table S1.

Table S1. Genes of *Alcaligenes faecalis* strain 13f and the other twelve *A. faecalis* strains included in phylogenomic analysis

Protein family number	Product
PGF_00008115	Glutamate synthase [NADPH] large chain (EC 1.4.1.13)
PGF_00425603	FAD/FMN-containing dehydrogenases
PGF_08152874	ATP-dependent helicase HrpA
PGF_03589837	CDA peptide synthetase I
PGF_00426722	5-methyltetrahydrofolate--homocysteine methyltransferase (EC 2.1.1.13)
PGF_10406880	DNA polymerase III alpha subunit (EC 2.7.7.7)
PGF_03057047	Multidrug efflux system, inner membrane proton/drug antiporter (RND type) => MexI of MexHI-OpmD system
PGF_05149076	Multidrug efflux system MdtABC-TolC, inner-membrane proton/drug antiporter MdtB (RND type)
PGF_01395856	RND efflux system, inner membrane transporter KPN_02144
PGF_10525419	Translation initiation factor 2
PGF_10461681	Ribonuclease E (EC 3.1.26.12)
PGF_09439882	Error-prone repair homolog of DNA polymerase III alpha subunit (EC 2.7.7.7)
PGF_04341022	Na(+) H(+) antiporter subunit A / Na(+) H(+) antiporter subunit B
PGF_00033392	Phosphoenolpyruvate carboxylase (EC 4.1.1.31)
PGF_10049811	Protein translocase subunit SecA
PGF_00008593	Ribosome-associated ATPase RbbA
PGF_01580528	Mg(2+) transport ATPase, P-type (EC 3.6.3.2)

Protein family number	Product
PGF_07830674	Alanyl-tRNA synthetase (EC 6.1.1.7)
PGF_10347756	DNA polymerase I (EC 2.7.7.7)
PGF_00019484	Membrane alanine aminopeptidase N (EC 3.4.11.2)
PGF_01053024	Glutamine synthetase adenylyl-L-tyrosine phosphorylase (EC 2.7.7.89) / Glutamate-ammonia-ligase adenylyltransferase (EC 2.7.7.42)
PGF_06812369	Leucyl-tRNA synthetase (EC 6.1.1.4)
PGF_02938414	Osmosensitive K+ channel histidine kinase KdpD
PGF_05562713	[Protein-PII] uridylyltransferase (EC 2.7.7.59) / [Protein-PII]-UMP uridylyl-removing enzyme
PGF_04438983	ATP-dependent protease La (EC 3.4.21.53) Type I
PGF_04962192	3'-to-5' exoribonuclease RNase R
PGF_05438553	Phosphoenolpyruvate synthase (EC 2.7.9.2)
PGF_00048630	Ribonucleotide reductase of class Ia (aerobic), alpha subunit (EC 1.17.4.1)
PGF_00010349	Guanosine-3',5'-bis(diphosphate) 3'-pyrophosphohydrolase (EC 3.1.7.2) / GTP pyrophosphokinase (EC 2.7.6.5), (p)ppGpp synthetase II
PGF_08155727	DNA translocase FtsK
PGF_10370701	Xanthine dehydrogenase, molybdenum binding subunit (EC 1.17.1.4)
PGF_00445746	hypothetical protein
PGF_09964285	LPS-assembly protein LptD @ Organic solvent tolerance protein precursor
PGF_03104485	Polyribonucleotide nucleotidyltransferase (EC 2.7.7.8)
PGF_00009969	Glycyl-tRNA synthetase beta chain (EC 6.1.1.14)
PGF_02226715	ATP-dependent DNA helicase RecG (EC 3.6.4.12)
PGF_03609651	Methionyl-tRNA synthetase (EC 6.1.1.10)
PGF_00019055	Maltodextrin glucosidase (EC 3.2.1.20)
PGF_00026904	Oligopeptidase A (EC 3.4.24.70)
PGF_01136362	Transketolase (EC 2.2.1.1)
PGF_00035406	Potassium-transporting ATPase B chain (EC 3.6.3.12) (TC 3.A.3.7.1)
PGF_00067569	Zinc-regulated outer membrane receptor

Protein family number	Product
PGF_09198321	General secretion pathway protein D
PGF_00979882	High-affinity choline uptake protein BetT
PGF_01175575	Threonyl-tRNA synthetase (EC 6.1.1.3)
PGF_00420496	Cytochrome c heme lyase subunit CcmF
PGF_06005188	DNA topoisomerase IV subunit B (EC 5.99.1.3)
PGF_06275407	Cell division-associated, ATP-dependent zinc metalloprotease FtsH
PGF_00576911	peptidyl-prolyl cis-trans isomerase D
PGF_00015707	Isocitrate dehydrogenase phosphatase (EC 2.7.11.5)/kinase (EC 3.1.3.-)
PGF_02756797	Putative peptidase
PGF_10376398	Peptidoglycan D,D-transpeptidase MrdA (EC 3.4.16.4)
PGF_00420952	3-oxoacyl-[acyl-carrier-protein] synthase III (EC 2.3.1.41)
PGF_01960322	Glutamine--fructose-6-phosphate aminotransferase [isomerizing] (EC 2.6.1.16)
PGF_05949521	Protein translocase subunit SecD
PGF_00007041	GTP-binding protein TypA/BipA
PGF_02030844	ATP-dependent DNA helicase RecQ
PGF_05875473	Efflux ABC transporter, permease/ATP-binding protein Atu2242
PGF_00005687	Flavin-containing monooxygenase
PGF_00426115	1-deoxy-D-xylulose 5-phosphate synthase (EC 2.2.1.7)
PGF_08147022	Chloride channel protein
PGF_00055049	Sulfite reductase [NADPH] flavoprotein alpha-component (EC 1.8.1.2)
PGF_09939762	SSU ribosomal protein S1p
PGF_00008326	Glutaminyl-tRNA synthetase (EC 6.1.1.18)
PGF_08515716	Lipid A export permease/ATP-binding protein MsbA
PGF_00066867	Xaa-Pro aminopeptidase (EC 3.4.11.9)
PGF_00055052	Sulfite reductase [NADPH] hemoprotein beta-component (EC 1.8.1.2)
PGF_08976331	Putative protease
PGF_00123288	Sulfate permease, Trk-type

Protein family number	Product
PGF_00018999	Malonate decarboxylase alpha subunit
PGF_00420992	D-lactate dehydrogenase (EC 1.1.1.28)
PGF_00408673	Ferredoxin-dependent glutamate synthase (EC 1.4.7.1)
PGF_03062842	Sulfate adenylyltransferase (EC 2.7.7.4) / Domain of unknown function
PGF_00035405	Potassium-transporting ATPase A chain (EC 3.6.3.12) (TC 3.A.3.7.1)
PGF_00416129	CTP synthase (EC 6.3.4.2)
PGF_00038267	Phenylacetic acid degradation protein PaaN2, ring-opening aldehyde dehydrogenase (EC 1.2.1.3)
PGF_00596539	Efflux ABC transporter for glutathione/L-cysteine, essential for assembly of bd-type respiratory oxidases => CydD subunit
PGF_03751973	Inner membrane protein translocase and chaperone YidC, long form
PGF_07501301	Sodium-dependent phosphate transporter
PGF_05424184	Na(+) H(+) antiporter subunit D
PGF_00033917	Acyl-coenzyme A synthetases/AMP-(fatty) acid ligases
PGF_03804538	Cytochrome c oxidase polypeptide I (EC 1.9.3.1)
PGF_08398438	FAD-dependent oxidoreductase SPO0682
PGF_00030643	Peptide chain release factor 3
PGF_00064536	Uncharacterized ABC1 family protein XCC_1720
PGF_00013509	IMP cyclohydrolase (EC 3.5.4.10) / Phosphoribosylaminoimidazolecarboxamide formyltransferase (EC 2.1.2.3)
PGF_09969389	Alkyl hydroperoxide reductase protein F
PGF_12719092	Paraquat-inducible protein B
PGF_00064460	Ubiquinone biosynthesis regulatory protein kinase UbiB
PGF_00024470	NAD-dependent formate dehydrogenase beta subunit
PGF_02325838	Apolipoprotein N-acyltransferase / Copper homeostasis protein CutE
PGF_02098099	Histidine ammonia-lyase (EC 4.3.1.3)
PGF_06041068	3-hydroxyacyl-CoA dehydrogenase (EC 1.1.1.35) / Enoyl CoA hydratase (EC 4.2.1.17)
PGF_00033014	Phenol hydroxylase, P3 oxygenase component DmpN (EC 1.14.13.7)

Protein family number	Product
PGF_07743758	Nucleoside ABC transporter, ATP-binding protein
PGF_00037588	Prolyl-tRNA synthetase (EC 6.1.1.15), bacterial type
PGF_06522349	Aspartyl-tRNA(Asn) amidotransferase subunit A (EC 6.3.5.6) @ Glutamyl-tRNA(Gln) amidotransferase subunit A (EC 6.3.5.7)
PGF_10444249	Murein hydrolase activator EnvC
PGF_00578001	hypothetical protein
PGF_07063065	Transcription termination protein NusA
PGF_12662797	Two-component system sensor histidine kinase
PGF_10048015	Inosine-5'-monophosphate dehydrogenase (EC 1.1.1.205) / CBS domain
PGF_00050995	Amidophosphoribosyltransferase (EC 2.4.2.14)
PGF_00420051	3-methylmercaptopropionyl-CoA ligase (EC 6.2.1.44) of DmdB1 type
PGF_06992864	Type cbb3 cytochrome oxidase biogenesis protein CcoG, involved in Cu oxidation
PGF_05471309	Exopolyphosphatase (EC 3.6.1.11)
PGF_04251132	Xanthine dehydrogenase iron-sulfur subunit (EC 1.17.1.4) / Xanthine dehydrogenase, FAD binding subunit (EC 1.17.1.4)
PGF_00497557	Uncharacterized protein YjgR
PGF_06707410	TldD protein, part of TldE/TldD proteolytic complex
PGF_00024322	NAD(P) transhydrogenase subunit beta (EC 1.6.1.2)
PGF_05195027	ATP synthase beta chain (EC 3.6.3.14)
PGF_03474542	Flagellum-specific ATP synthase FliI
PGF_08755123	dolichyl-phosphate-mannose-protein mannosyltransferase family protein
PGF_07760799	Pyruvate kinase (EC 2.7.1.40)
PGF_00040673	Putative drug efflux protein
PGF_06196148	D-serine/D-alanine/glycine transporter
PGF_00419628	Coproporphyrinogen III oxidase, oxygen-independent (EC 1.3.99.22)
PGF_02064356	Adenosylhomocysteinase (EC 3.3.1.1)
PGF_08562657	tRNA-i(6)A37 methylthiotransferase (EC 2.8.4.3)

Protein family number	Product
PGF_12698796	Ubiquinol-cytochrome C reductase, cytochrome B subunit (EC 1.10.2.2)
PGF_00264649	hypothetical protein
PGF_00280580	hypothetical protein
PGF_00008335	Glutamyl-tRNA synthetase (EC 6.1.1.17)
PGF_04425336	NADH-ubiquinone oxidoreductase chain F (EC 1.6.5.3)
PGF_00043931	Allantoinase (EC 3.5.2.5)
PGF_08109371	Membrane-bound lytic murein transglycosylase D
PGF_05940986	D-beta-hydroxybutyrate permease
PGF_08745438	tRNA/rRNA cytosine-C5-methylase
PGF_00010337	Guanine deaminase (EC 3.5.4.3); Hydroxydechloroatrazine ethylaminohydrolase (EC 3.5.99.3)
PGF_00034566	Poly(A) polymerase (EC 2.7.7.19)
PGF_00007024	GTP-binding protein EngA
PGF_06162930	UDP-N-acetylmuramoyl-L-alanine--D-glutamate ligase (EC 6.3.2.9)
PGF_07075462	Periplasmic chaperone and peptidyl-prolyl cis-trans isomerase of outer membrane proteins SurA (EC 5.2.1.8)
PGF_08454293	N-acetylglucosamine-1-phosphate uridyltransferase (EC 2.7.7.23) / Glucosamine-1-phosphate N-acetyltransferase (EC 2.3.1.157)
PGF_00019038	ATP-dependent hsl protease ATP-binding subunit HslU
PGF_00577113	hypothetical protein
PGF_02620298	Argininosuccinate lyase (EC 4.3.2.1)
PGF_00033074	Phenylalanine-specific permease
PGF_02862285	Argininosuccinate synthase (EC 6.3.4.5)
PGF_00048846	Ribosomal protein S12p Asp88 (E. coli) methylthiotransferase (EC 2.8.4.4)
PGF_12812074	Dihydrolipoamide dehydrogenase (EC 1.8.1.4)
PGF_05837052	Isocitrate lyase (EC 4.1.3.1)
PGF_10535181	Tol-Pal system beta propeller repeat protein TolB
PGF_00766022	Bis-ABC ATPase YbiT

Protein family number	Product
PGF_06945838	Na+/H+ antiporter NhaA type
PGF_00423086	Diaminobutyrate--2-oxoglutarate transaminase (EC 2.6.1.76)
PGF_00066906	Aspartate carbamoyltransferase (EC 2.1.3.2)
PGF_01054379	Adenylosuccinate lyase (EC 4.3.2.2) @ SAICAR lyase (EC 4.3.2.2)
PGF_00414710	Pyrimidine permease
PGF_00492707	Homoserine dehydrogenase (EC 1.1.1.3)
PGF_00064004	UDP-N-acetylmuramate:L-alanyl-gamma-D-glutamyl-meso-diaminopimelate ligase (EC 6.3.2.-)
PGF_00947361	Phenylacetate-coenzyme A ligase (EC 6.2.1.30)
PGF_00010332	Guanine deaminase (EC 3.5.4.3)
PGF_02452671	Cell division trigger factor (EC 5.2.1.8)
PGF_05387084	Gamma-glutamyl phosphate reductase (EC 1.2.1.41)
PGF_07186360	NAD-specific glutamate dehydrogenase (EC 1.4.1.2); NADP-specific glutamate dehydrogenase (EC 1.4.1.4)
PGF_00006510	Fumarylacetoacetase (EC 3.7.1.2)
PGF_01781129	Miniconductance mechanosensitive channel YbdG
PGF_10393859	Long-chain fatty acid transport protein
PGF_00008334	Glutamyl-tRNA reductase (EC 1.2.1.70)
PGF_02617052	Transcriptional regulator of pyridoxine metabolism / Pyridoxamine phosphate aminotransferase (EC 2.6.1.54)
PGF_00012291	Homogentisate 1,2-dioxygenase (EC 1.13.11.5)
PGF_00769755	16S rRNA (cytosine(967)-C(5))-methyltransferase (EC 2.1.1.176)
PGF_00042508	Putative outer membrane protein
PGF_10452187	Phosphoglucomutase (EC 5.4.2.2) @ Phosphomannomutase (EC 5.4.2.8)
PGF_00015701	Isocitrate dehydrogenase [NADP] (EC 1.1.1.42)
PGF_00576870	hypothetical protein
PGF_07093307	UDP-N-acetyl-D-glucosamine 6-dehydrogenase (EC 1.1.1.136)
PGF_00054959	Sulfate adenylyltransferase subunit 1 (EC 2.7.7.4)

Protein family number	Product
PGF_07844318	3-phosphoshikimate 1-carboxyvinyltransferase (EC 2.5.1.19)
PGF_03004613	Histidyl-tRNA synthetase (EC 6.1.1.21)
PGF_06935032	Adenylosuccinate synthetase (EC 6.3.4.4)
PGF_00409934	putative oxygenase subunit
PGF_10373476	Diaminopimelate decarboxylase (EC 4.1.1.20)
PGF_07583562	Cysteine desulfurase (EC 2.8.1.7) => SufS
PGF_00063916	Tyrosyl-tRNA synthetase (EC 6.1.1.1)
PGF_00580333	Lipopolysaccharide assembly protein LapB
PGF_01147190	Deoxyribodipyrimidine photolyase (EC 4.1.99.3)
PGF_00011634	HflK protein
PGF_03448459	S-adenosylmethionine decarboxylase proenzyme (EC 4.1.1.50), prokaryotic class 1B
PGF_00020168	Membrane-bound lytic murein transglycosylase A
PGF_00576920	hypothetical protein
PGF_06614457	Ammonium transporter
PGF_00062027	Tryptophan synthase beta chain (EC 4.2.1.20)
PGF_00003526	FIG137887: membrane protein related to purine degradation
PGF_00400845	Uncharacterized integral membrane endopeptidase Bmul_2226
PGF_00094331	hypothetical protein
PGF_01044980	putative membrane protein
PGF_05498463	Glutamate-pyruvate aminotransferase AlaC (EC 2.6.1.2)
PGF_00014284	Inner membrane transport protein YajR
PGF_07941512	23S rRNA (uracil(1939)-C(5))-methyltransferase (EC 2.1.1.190)
PGF_02944756	3-ketoacyl-CoA thiolase [fadN-fadA-fadE operon] (EC 2.3.1.16)
PGF_00017109	Lipid-A-disaccharide synthase (EC 2.4.1.182)
PGF_00027514	N-acetylornithine aminotransferase (EC 2.6.1.11)
PGF_00007022	GTP-binding protein EngA
PGF_02277678	Phosphoglycerate kinase (EC 2.7.2.3)

Protein family number	Product
PGF_00030070	Patatin
PGF_10125426	Peptidoglycan glycosyltransferase FtsW (EC 2.4.1.129)
PGF_00403929	Biosynthetic Aromatic amino acid aminotransferase alpha (EC 2.6.1.57)
PGF_00032869	Isovaleryl-CoA dehydrogenase (EC 1.3.8.4)
PGF_00033359	Phospho-N-acetylmuramoyl-pentapeptide-transferase (EC 2.7.8.13)
PGF_00055902	TRAP-type transport system, large permease component, predicted N-acetylneuraminate transporter
PGF_03520151	Cell division protein FtsZ
PGF_03202156	S-adenosylmethionine synthetase (EC 2.5.1.6)
PGF_00000939	Uncharacterized protein YhiN
PGF_09111052	Phosphopantothenoylcysteine decarboxylase (EC 4.1.1.36) / Phosphopantothenoylcysteine synthetase (EC 6.3.2.5)
PGF_07459509	L-aspartate oxidase (EC 1.4.3.16)
PGF_00007353	General secretion pathway protein F
PGF_00054528	Succinyl-CoA ligase [ADP-forming] beta chain (EC 6.2.1.5)
PGF_00025679	Acetyl-CoA acetyltransferase (EC 2.3.1.9) @ 3-oxoadipyl-CoA thiolase (EC 2.3.1.174)
PGF_00067277	Valine--pyruvate aminotransferase (EC 2.6.1.66)
PGF_09794316	Glutamate/aspartate ABC transporter, permease protein GltJ (TC 3.A.1.3.4)
PGF_02098447	SAM-dependent methyltransferase, MidA
PGF_09129813	Glutamate 5-kinase (EC 2.7.2.11) / RNA-binding C-terminal domain PUA
PGF_00420081	Cysteine synthesis adenylyltransferase/sulfurtransferase
PGF_00024274	N5-carboxyaminoimidazole ribonucleotide synthase (EC 6.3.4.18)
PGF_00043489	Possible regulatory protein similar to urea ABC transporter, substrate binding protein
PGF_05292270	Ribonucleotide reductase of class Ia (aerobic), beta subunit (EC 1.17.4.1)
PGF_00073653	hypothetical protein
PGF_09031367	Benzoate 1,2-dioxygenase alpha subunit (EC 1.14.12.10)
PGF_04965976	Putrescine transport ATP-binding protein PotG (TC 3.A.1.11.2)

Protein family number	Product
PGF_03006190	2-polyprenyl-6-methoxyphenol hydroxylase
PGF_02516666	Cytochrome c oxidase polypeptide II (EC 1.9.3.1)
PGF_00007511	Gentisate 1,2-dioxygenase (EC 1.13.11.4)
PGF_00051358	Serine-pyruvate aminotransferase/archaeal aspartate aminotransferase
PGF_02186693	Flagellar biosynthesis protein FlhB
PGF_00014838	Integrase
PGF_00037374	Probable sulfite reductase
PGF_02061452	Queuine tRNA-ribosyltransferase (EC 2.4.2.29)
PGF_09945671	Acetate kinase (EC 2.7.2.1)
PGF_02963785	NnrS protein involved in response to NO
PGF_00423089	Diaminohydroxyphosphoribosylaminopyrimidine deaminase (EC 3.5.4.26) / 5-amino-6-(5-phosphoribosylamino)uracil reductase (EC 1.1.1.193)
PGF_00063948	UDP-3-O-[3-hydroxymyristoyl] glucosamine N-acyltransferase (EC 2.3.1.191)
PGF_12764040	Deoxyguanosinetriphosphate triphosphohydrolase (EC 3.1.5.1)
PGF_04284526	Alpha-aminoadipate aminotransferase (EC 2.6.1.39) @ Leucine transaminase (EC 2.6.1.6) @ Valine transaminase
PGF_00070354	2-keto-3-deoxy-D-arabino-heptulosonate-7-phosphate synthase I alpha (EC 2.5.1.54)
PGF_00441123	hypothetical protein
PGF_05696702	Sulfate and thiosulfate import ATP-binding protein CysA (EC 3.6.3.25)
PGF_10443448	Pyrimidine monooxygenase-like protein
PGF_00576895	hypothetical protein
PGF_00027236	Opine dehydrogenase (EC 1.5.1.28)
PGF_00067739	[4Fe-4S] cluster assembly scaffold protein Mrp (=ApbC)
PGF_05866109	Outer membrane beta-barrel assembly protein BamC
PGF_00030640	Peptide chain release factor 1
PGF_00007028	Ribosome LSU-associated GTP-binding protein HflX
PGF_03811905	Histidinol-phosphate aminotransferase (EC 2.6.1.9)

Protein family number	Product
PGF_00024992	NICKEL-COBALT-CADMIUM RESISTANCE PROTEIN NCCN
PGF_10143857	Fructose-bisphosphate aldolase class II (EC 4.1.2.13)
PGF_00353488	hypothetical protein
PGF_09188652	Molybdopterin molybdenumtransferase (EC 2.10.1.1)
PGF_08461341	Lipopolysaccharide export system permease protein LptF
PGF_07889681	N-acetyl-gamma-glutamyl-phosphate reductase (EC 1.2.1.38)
PGF_05346766	TolA protein
PGF_00005450	Flagellar hook protein FlgE
PGF_02390924	16S rRNA (cytosine(1402)-N(4))-methyltransferase (EC 2.1.1.199)
PGF_07015581	Carbamoyl-phosphate synthase small chain (EC 6.3.5.5)
PGF_03295331	UDP-N-acetylglucosamine--N-acetylmuramyl-(pentapeptide) pyrophosphoryl-undecaprenol N-acetylglucosamine transferase (EC 2.4.1.227)
PGF_00033005	Phenol hydroxylase, FAD- and [2Fe-2S]-containing reductase component DmpP
PGF_03057995	Glycosyl transferase, group 1 family protein
PGF_00688906	Nucleoside ABC transporter, permease protein 1
PGF_01059886	Hydroxymethylpyrimidine ABC transporter, substrate-binding component
PGF_00696203	Peptide chain release factor 2 @ programmed frameshift-containing
PGF_00423382	Dihydroorotase (EC 3.5.2.3)
PGF_07668761	Phosphate ABC transporter, substrate-binding protein PstS (TC 3.A.1.7.1)
PGF_00008611	Glycerol-3-phosphate dehydrogenase [NAD(P)+] (EC 1.1.1.94)
PGF_00055809	TRAP transporter solute receptor, unknown substrate 7
PGF_03439803	Para-aminobenzoate synthase, aminase component (EC 2.6.1.85) / Aminodeoxychorismate lyase (EC 4.1.3.38)
PGF_01400330	Biotin synthase (EC 2.8.1.6)
PGF_01900675	Flagellar motor switch protein FliM
PGF_08058915	hypothetical protein
PGF_03116630	Membrane-bound lytic murein transglycosylase B

Protein family number	Product
PGF_01033770	Dihydroorotate dehydrogenase (quinone) (EC 1.3.5.2)
PGF_01867628	Heat-inducible transcription repressor HrcA
PGF_06755829	DNA polymerase III delta prime subunit (EC 2.7.7.7)
PGF_00489714	Porphobilinogen synthase (EC 4.2.1.24)
PGF_00419215	hypothetical protein
PGF_00035194	Adenosine deaminase (EC 3.5.4.4)
PGF_00880281	hypothetical protein
PGF_00394669	RND efflux system, membrane fusion protein KPN_02145
PGF_00021489	Uncharacterized membrane protein Bcep18194_A6058
PGF_00052053	Sigma factor RpoE negative regulatory protein RseB precursor
PGF_00667785	Phenol hydroxylase, P1 oxygenase component DmpL (EC 1.14.13.7)
PGF_00911329	Acryloyl-CoA reductase AcuI/YhdH (EC 1.3.1.84)
PGF_01456294	3-hydroxybutyryl-CoA dehydrogenase (EC 1.1.1.157)
PGF_03971607	Iron(III) dicitrate transport system permease protein FecD (TC 3.A.1.14.1)
PGF_07191648	3-isopropylmalate dehydrogenase (EC 1.1.1.85)
PGF_00003448	FIG110192: hypothetical protein
PGF_09112178	Threonylcarbamoyl-AMP synthase (EC 2.7.7.87) / SUA5 domain with internal deletion
PGF_00420953	3-oxoacyl-[acyl-carrier-protein] synthase, KASIII (EC 2.3.1.180)
PGF_00047206	2-amino-3-carboxymuconate-6-semialdehyde decarboxylase (EC 4.1.1.45)
PGF_00050368	Selenophosphate-dependent tRNA 2-selenouridine synthase
PGF_05399159	Acetyl-coenzyme A carboxyl transferase alpha chain (EC 6.4.1.2)
PGF_02623406	FMN adenylyltransferase (EC 2.7.7.2) / Riboflavin kinase (EC 2.7.1.26)
PGF_00420903	D-arabinose-5-phosphate isomerase (EC 5.3.1.13)
PGF_04835795	Site-specific tyrosine recombinase XerC
PGF_02904832	Homoserine kinase (EC 2.7.1.39)
PGF_10363729	Alkanesulfonate utilization operon LysR-family regulator Cbl
PGF_00405994	putative N-oxidase

Protein family number	Product
PGF_10569727	LSU rRNA pseudouridine(1911/1915/1917) synthase (EC 5.4.99.23)
PGF_08244241	Tetraacyldisaccharide 4'-kinase (EC 2.7.1.130)
PGF_00423732	4-hydroxy-3-methylbut-2-enyl diphosphate reductase (EC 1.17.7.4)
PGF_03758468	Meso-diaminopimelate D-dehydrogenase (EC 1.4.1.16)
PGF_00576871	hypothetical protein
PGF_00063430	Type II/IV secretion system protein TadC, associated with Flp pilus assembly
PGF_00776546	hypothetical protein
PGF_10346917	hypothetical protein
PGF_08180671	hypothetical protein
PGF_00912265	tRNA-dihydrouridine synthase DusB
PGF_00063944	UDP-3-O-[3-hydroxymyristoyl] N-acetylglucosamine deacetylase (EC 3.5.1.108)
PGF_00057399	Transaldolase (EC 2.2.1.2)
PGF_10551687	hypothetical protein
PGF_00063974	UDP-N-acetylenolpyruvoylglucosamine reductase (EC 1.3.1.98)
PGF_00066237	Uricase (urate oxidase) (EC 1.7.3.3)
PGF_04645019	HPr kinase/phosphorylase
PGF_00709627	Nucleoside ABC transporter, permease protein 2
PGF_00026866	Octaprenyl diphosphate synthase (EC 2.5.1.90)
PGF_00005572	Flagellar protein FlgJ [peptidoglycan hydrolase]
PGF_03739662	FIG00432062: Rhodanese-related sulfurtransferase
PGF_10462808	33 kDa chaperonin HslO
PGF_00016046	L-asparaginase (EC 3.5.1.1)
PGF_05165078	Methionyl-tRNA formyltransferase (EC 2.1.2.9)
PGF_05672678	Beta-propeller domains of methanol dehydrogenase type
PGF_06784545	(2E,6E)-farnesyl diphosphate synthase (EC 2.5.1.10)
PGF_00045738	Putrescine transport system permease protein PotH (TC 3.A.1.11.2)
PGF_06655223	Malonyl CoA-acyl carrier protein transacylase (EC 2.3.1.39)

Protein family number	Product
PGF_02104569	Cysteine synthase B (EC 2.5.1.47)
PGF_00026615	N-acetylglutamate kinase (EC 2.7.2.8)
PGF_00393545	Uncharacterized sodium-dependent transporter YocS
PGF_00048840	Ribosomal protein L11 methyltransferase
PGF_03815442	Cytochrome c oxidase (cbb3-type) subunit CcoP (EC 1.9.3.1)
PGF_03364965	Type cbb3 cytochrome oxidase biogenesis protein CcoI; Copper-translocating P-type ATPase (EC 3.6.3.4)
PGF_00048842	Ribosomal protein L3 N(5)-glutamine methyltransferase (EC 2.1.1.298)
PGF_03701810	Cell-division-associated, ABC-transporter-like signaling protein FtsX
PGF_09221959	Methylisocitrate lyase (EC 4.1.3.30)
PGF_00019595	Membrane protease family protein y2843
PGF_06053106	RNA polymerase sigma factor RpoH
PGF_06331725	Integral membrane protein
PGF_04150742	Zinc ABC transporter, permease protein ZnuB
PGF_00007027	GTP-binding protein Era
PGF_02191019	Thiamine-monophosphate kinase (EC 2.7.4.16)
PGF_00019015	Malonate utilization transcriptional regulator
PGF_00017281	Lipopolysaccharide core heptosyltransferase I
PGF_08518355	ATP synthase F0 sector subunit a (EC 3.6.3.14)
PGF_00419627	Coproporphyrinogen III oxidase, aerobic (EC 1.3.3.3)
PGF_01189572	Triphosphoribosyl-dephospho-CoA synthase (EC 2.4.2.52)
PGF_04807486	tRNA dimethylallyltransferase (EC 2.5.1.75)
PGF_00020996	Methylglyoxal synthase (EC 4.2.3.3)
PGF_10525969	Magnesium and cobalt efflux protein CorC
PGF_05785543	Signal peptidase I (EC 3.4.21.89)
PGF_00037828	Protease HtpX
PGF_00577080	hypothetical protein

Protein family number	Product
PGF_02903501	Glycerol-3-phosphate ABC transporter, permease protein UgpA (TC 3.A.1.1.3)
PGF_05091456	Uncharacterized inner membrane protein RarD
PGF_03010412	ABC transporter, permease protein PA3837
PGF_10480638	Cytochrome c oxidase polypeptide III (EC 1.9.3.1)
PGF_05481950	hypothetical protein
PGF_06925665	ATPase associated with various cellular activities, AAA_5
PGF_00151525	hypothetical protein
PGF_00019000	Malonate decarboxylase beta subunit
PGF_00244553	hypothetical protein
PGF_03870438	Uncharacterized peptidase U32 family member YhbV
PGF_00652709	hypothetical protein
PGF_03124227	Pyruvate:Oxaloacetate transcarboxylase domain protein
PGF_00376758	ATP/GTP-binding protein
PGF_00122014	hypothetical protein
PGF_00020984	Methenyltetrahydrofolate cyclohydrolase (EC 3.5.4.9) / Methylenetetrahydrofolate dehydrogenase (NADP+) (EC 1.5.1.5)
PGF_00006351	Efflux ABC transporter, permease protein
PGF_02334240	Biotin--protein ligase (EC 6.3.4.9)(EC 6.3.4.10)(EC 6.3.4.11)(EC 6.3.4.15)
PGF_07260646	Ectoine/hydroxyectoine ABC transporter solute-binding protein, EhuB
PGF_02323834	Cyclohexadienyl dehydrogenase (EC 1.3.1.12)(EC 1.3.1.43)
PGF_03198057	Inner membrane protein YpjD
PGF_04547504	hypothetical protein
PGF_08154601	Glycerol-3-phosphate ABC transporter, permease protein UgpE (TC 3.A.1.1.3)
PGF_10538695	Permease of the drug/metabolite transporter (DMT) superfamily
PGF_00016393	LSU ribosomal protein L2p (L8e)
PGF_00004127	Fe(2+)/alpha-ketoglutarate-dependent dioxygenase LpxO
PGF_00415130	23S rRNA (adenine(1618)-N(6))-methyltransferase (EC 2.1.1.181)

Protein family number	Product
PGF_00002514	FIG016425: Soluble lytic murein transglycosylase and related regulatory proteins (some contain LysM/invasin domains)
PGF_01853493	Dihydropteroate synthase (EC 2.5.1.15)
PGF_00084324	Undecaprenyl-diphosphatase (EC 3.6.1.27)
PGF_04321257	Flagellar motor rotation protein MotA
PGF_00064393	UTP--glucose-1-phosphate uridylyltransferase (EC 2.7.7.9)
PGF_00020172	2,3,4,5-tetrahydropyridine-2,6-dicarboxylate N-succinyltransferase (EC 2.3.1.117)
PGF_00595771	Squalene synthase (EC 2.5.1.21)
PGF_00062023	Tryptophan synthase alpha chain (EC 4.2.1.20)
PGF_00180261	hypothetical protein
PGF_00025136	NUDIX hydrolase, associated with Thiamin pyrophosphokinase
PGF_10354267	hypothetical protein
PGF_00577094	hypothetical protein
PGF_03843396	Glutamyl-Q tRNA(Asp) synthetase
PGF_10363383	Murein hydrolase activator NlpD
PGF_05580933	Peptide chain release factor N(5)-glutamine methyltransferase (EC 2.1.1.297)
PGF_07015776	Small-conductance mechanosensitive channel
PGF_05872952	Outer membrane beta-barrel assembly protein BamD
PGF_02473279	tRNA (cytidine(32)/uridine(32)-2'-O)-methyltransferase (EC 2.1.1.200)
PGF_07609122	Pyrroline-5-carboxylate reductase (EC 1.5.1.2)
PGF_00576875	hypothetical protein
PGF_00409679	putative membrane protein
PGF_00571298	Histidine, proline, and proline betaine ABC transporter, substrate-binding protein HutV
PGF_05740384	Formamidopyrimidine-DNA glycosylase (EC 3.2.2.23)
PGF_06889881	Flagellar motor rotation protein MotA
PGF_04803924	Putrescine transport system permease protein PotI (TC 3.A.1.11.2)

Protein family number	Product
PGF_00419915	3-methyl-2-oxobutanoate hydroxymethyltransferase (EC 2.1.2.11)
PGF_07763915	Bis(5'-nucleosyl)-tetraphosphatase, symmetrical (EC 3.6.1.41)
PGF_00033729	Acyl-[acyl-carrier-protein]--UDP-N-acetylglucosamine O-acyltransferase (EC 2.3.1.129)
PGF_08376928	Branched-chain amino acid aminotransferase (EC 2.6.1.42)
PGF_02781328	NAD synthetase (EC 6.3.1.5)
PGF_03846068	Pyridoxal kinase (EC 2.7.1.35)
PGF_07114837	Phospholipid ABC transporter permease protein MlaE
PGF_10300691	ABC transporter, ATP-binding protein (cluster 8, B12/iron complex)
PGF_00049889	SSU ribosomal protein S3p (S3e)
PGF_02486076	Zinc ABC transporter, substrate-binding protein ZnuA
PGF_09655255	Transcriptional regulator, RpiR family
PGF_00226685	hypothetical protein
PGF_00906364	Hydroxyacylglutathione hydrolase (EC 3.1.2.6)
PGF_01900676	ABC transporter, ATP-binding protein (cluster 9, phospholipid)
PGF_00357420	hypothetical protein
PGF_00024855	NADPH-dependent 7-cyano-7-deazaguanine reductase (EC 1.7.1.13)
PGF_07498099	Peptidyl-prolyl cis-trans isomerase (EC 5.2.1.8)
PGF_00041289	Putative hemolysin
PGF_01922821	Zn-dependent protease with chaperone function PA4632
PGF_00761827	Beta-ketoadipate enol-lactone hydrolase (EC 3.1.1.24)
PGF_01176589	4-hydroxy-tetrahydrodipicolinate reductase (EC 1.17.1.8)
PGF_00013266	ADP-ribose pyrophosphatase of COG1058 family (EC 3.6.1.13)
PGF_10373609	CDP-diacylglycerol--serine O-phosphatidyltransferase (EC 2.7.8.8)
PGF_03790040	Ribonuclease III (EC 3.1.26.3)
PGF_00064270	UPF0246 protein YaaA
PGF_00041524	Alkanesulfonates transport system permease protein
PGF_00260989	hypothetical protein

Protein family number	Product
PGF_02935470	UPF0317 protein YcsI
PGF_00011803	Histidine ABC transporter, ATP-binding protein HisP (TC 3.A.1.3.1)
PGF_06296193	N-formylglutamate deformylase (EC 3.5.1.68)
PGF_04720541	Glutamine ABC transporter, substrate-binding protein GlnH
PGF_05624364	4-(hydroxymethyl)benzenesulfonate dehydrogenase (EC 1.1.1.257)
PGF_05318435	Electron transfer flavoprotein, beta subunit
PGF_04213876	Pantothenate kinase type III, CoaX-like (EC 2.7.1.33)
PGF_07668817	Ferredoxin--NADP(+) reductase (EC 1.18.1.2) @ Flavodoxin--NADP(+) reductase (EC 1.19.1.1)
PGF_00577085	hypothetical protein
PGF_04505269	SSU ribosomal protein S2p (SAe)
PGF_09926890	ABC transporter, permease protein (cluster 10, nitrate/sulfonate/bicarbonate)
PGF_07952988	1,2-phenylacetyl-CoA epoxidase, subunit C (EC 1.14.13.149)
PGF_04127376	Twin-arginine translocation protein TatC
PGF_04710902	Lactam utilization protein LamB
PGF_00048586	Ribonuclease PH (EC 2.7.7.56)
PGF_00984073	Phosphate transport system regulatory protein PhoU
PGF_00051120	Septum site-determining protein MinC
PGF_09952093	Cytochrome oxidase biogenesis protein Surf1, facilitates heme A insertion
PGF_00042168	Putative metalloprotease yggG (EC 3.4.24.-)
PGF_06326847	Flagellar biosynthesis protein FliP
PGF_00418128	3-deoxy-manno-octulosonate cytidylyltransferase (EC 2.7.7.38)
PGF_00030017	Paralog of coenzyme PQQ synthesis protein C
PGF_05572422	Molybdenum ABC transporter, substrate-binding protein ModA
PGF_00040580	Putative deoxyribonuclease YjjV
PGF_01032153	Putative uncharacterized protein BCG_3011c
PGF_00413208	tRNA (guanine(37)-N(1))-methyltransferase (EC 2.1.1.228)
PGF_02923127	Uridylate kinase (EC 2.7.4.22)

Protein family number	Product
PGF_00426342	FIG005121: SAM-dependent methyltransferase (EC 2.1.1.-)
PGF_00417755	3-demethylubiquinol 3-O-methyltransferase (EC 2.1.1.64) @ 2-polyprenyl-6-hydroxyphenyl methylase (EC 2.1.1.222)
PGF_00083594	hypothetical protein
PGF_00340056	hypothetical protein
PGF_00721215	Zinc ABC transporter, ATP-binding protein ZnuC
PGF_04574228	Triosephosphate isomerase (EC 5.3.1.1)
PGF_00425810	Cell division protein ZapD
PGF_00214580	hypothetical protein
PGF_02992100	Arginyl-tRNA--protein transferase (EC 2.3.2.8)
PGF_06649360	Segregation and condensation protein B
PGF_02866563	Pyridoxal-5'-phosphate phosphatase (EC 3.1.3.74), Alphaproteobacterial type
PGF_00974916	Histidine ABC transporter, permease protein HisM (TC 3.A.1.3.1)
PGF_00406665	putative decarboxylase
PGF_00576925	hypothetical protein
PGF_00577185	hypothetical protein
PGF_00063937	UDP-2,3-diacylglucosamine diphosphatase (EC 3.6.1.54)
PGF_04776928	Glycerophosphoryl diester phosphodiesterase (EC 3.1.4.46)
PGF_06269104	Monofunctional biosynthetic peptidoglycan transglycosylase
PGF_00045510	Two-component system response regulator protein
PGF_02246769	Ectoine/hydroxyectoine ABC transporter permease protein, EhuD
PGF_00420482	Cytochrome c-type biogenesis protein CcmC, putative heme lyase for CcmE
PGF_00011816	Histidine ABC transporter, permease protein HisQ (TC 3.A.1.3.1)
PGF_03751326	Epoxyqueuosine reductase (EC 1.17.99.6) QueH
PGF_08105114	hypothetical protein
PGF_00118498	hypothetical protein
PGF_10512754	Fatty acid hydroxylase family (carotene hydroxylase/sterol desaturase)
PGF_00392956	hypothetical protein

Protein family number	Product
PGF_10245672	Ribulose-phosphate 3-epimerase (EC 5.1.3.1)
PGF_02489591	Uncharacterized Nudix hydrolase NudL
PGF_00798644	SAM-dependent methyltransferase
PGF_00648054	Tol-Pal system protein TolQ
PGF_00019004	Malonate decarboxylase gamma subunit
PGF_00024615	NADH pyrophosphatase (EC 3.6.1.22), decaps 5'-NAD modified RNA
PGF_08607235	Flagellar L-ring protein FlgH
PGF_00260861	hypothetical protein
PGF_05501316	FIG00003370: Multicopper polyphenol oxidase
PGF_09015297	Two-component response regulatory protein BP2547
PGF_00695496	Succinyl-CoA:3-ketoacid-coenzyme A transferase subunit A (EC 2.8.3.5)
PGF_00577079	hypothetical protein
PGF_00016431	LSU ribosomal protein L3p (L3e)
PGF_00565351	ABC transporter involved in cytochrome c biogenesis, CcmB subunit
PGF_00777996	5'-methylthioadenosine nucleosidase (EC 3.2.2.16) @ S-adenosylhomocysteine nucleosidase (EC 3.2.2.9)
PGF_00662510	Aldo/keto reductase
PGF_00160399	hypothetical protein
